# A prospective, quantitative study of mental health act assessments in England following the 2007 amendments to the 1983 act: did the changes fulfill their promise?

**DOI:** 10.1186/s12888-017-1391-2

**Published:** 2017-07-10

**Authors:** Swaran P. Singh, Moli Paul, Helen Parsons, Tom Burns, Peter Tyrer, Seena Fazel, Shoumitro Deb, Zoebia Islam, Jorun Rugkåsa, Ruchika Gajwani, Lavanya Thana, Michael J. Crawford

**Affiliations:** 1 Mental Health and Wellbeing, Warwick Medical School, Coventry, UK; 2 Division of Health Sciences, Warwick Medical School, Coventry, UK; 3 University of Oxford, Warneford Hospital, Oxford, UK; 4 Imperial College London, Centre for Psychiatry, Division of Brain Sciences, Department of Medicine, London, UK; 5 LOROS (The Leicestershire and Rutland Hospice), Leicester, UK; 6 Health Services Research Unit, Akershus University Hospital, Akershus, Norway; 7 Institute of Health and Wellbeing, University of Glasgow, Glasgow, UK; 8 Stratford CAMHS, Coventry and Warwickshire Partnership Trust, Stratford-upon-Avon, UK

**Keywords:** Psychiatry, Mental health, Law, Act, Involuntary

## Abstract

**Background:**

In 2008, the Mental Health Act (MHA) 2007 amendments to the MHA 1983 were implemented in England and Wales. The amendments were intended to remove perceived obstacles to the detention of high risk patients with personality disorders (PDs), sexual deviance and learning disabilities (LDs). The AMEND study aimed to test the hypothesis that the implementation of these changes would lead to an increase in numbers or proportions of patients with these conditions who would be assessed and detained under the MHA 2007.

**Method:**

A prospective, quantitative study of MHA assessments undertaken between July–October 2008–11 at three English sites. Data were collected from local forms used for MHA assessment documentation and patient electronic databases.

**Results:**

The total number of assessments in each four month period of data collection varied: 1034 in 2008, 1042 in 2009, 1242 in 2010 and 1010 in 2011 (*n* = 4415). Of the assessments 65.6% resulted in detention in 2008, 71.3% in 2009, 64.7% in 2010 and 63.5% in 2011. There was no significant change in the odds ratio of detention when comparing the 2008 assessments against the combined 2009, 2010 and 2011 data (OR = 1.025, Fisher‘s exact *Χ* ^*2*^ *p* = 0.735). Only patients with LD and ‘any other disorder or disability of the mind’ were significantly more likely to be assessed under the MHA post implementation (Χ^2^ = 5.485, *P* = 0.018; Χ^2^ = 24.962, *P* > 0.001 respectively). There was no significant change post implementation in terms of the diagnostic category of detained patients.

**Conclusions:**

In the first three years post implementation, the 2007 Act did not facilitate the compulsory care of patients with PDs, sexual deviance and LDs.

## Background

On 3rd November 2008 the Mental Health Act (MHA) 2007 was implemented in England and Wales, amending the MHA 1983 [1, 2]. Amongst other changes, the 2007 Act enabled supervised community treatment and broadened the range of professionals who could apply for the detention of patients and fulfil the Responsible Clinician role. It substituted a single definition of *mental disorder* for the previous four categories (*mental illness*, *mental impairment*, *severe mental impairment* and *psychopathic disorder*) in the 1983 Act. It also abolished the so-called “treatability test” associated with the 1983 Act which had often been used to exclude compulsory treatment of those with conditions thought to be ‘untreatable’. These changes were made in an effort to ensure that individuals with mental disorders such as learning disabilities (LDs) and personality disorders (PDs), who might have previously been thought of as ‘untreatable’, and those with sexual deviance (paraphilias were specifically excluded in the MHA 1983) were not denied treatment.

AMEND is the first study of a large cohort of patients assessed under the MHA, over four years around the implementation of the 2007 amendments to the 1983 Act, from several sites. It follows the drive to study the impact of ‘new’ mental health legislation, as happened following the MHAs 1959 [3] and 1983 [4] and studies on legislative changes in other countries, especially those that led to a significant change in the delivery of mental health care [5]. The overall aim of the multi-stage AMEND study was to explore the extent to which these changes made an impact on clinical practice, user experience and service availability (full report available on request). This paper presents the results of the main quantitative study, designed to test the hypotheses that there would be a significant increases in the overall numbers of detentions and, specifically, detention of patients with PDs, sexual deviance, LDs or developmental disorders after the introduction of the 2007 MHA. The relationship between ethnicity and the detention has previously been reported [6].

## Methods

This was a prospective study of MHA assessments before and after implementation of the MHA 2007 in England and Wales. The AMEND study was conducted across three sites: West Midlands (Birmingham & Solihull Mental Health Trust - BSMHFT), south of England (Oxfordshire and Buckinghamshire Mental Health Foundation Trust – OBMHFT) and London (Central and North West London National Health Service (NHS) Foundation Trust - CNWL - and West London Mental Health Trust - WLMHT). There was heterogeneity in the socio-economic and ethnic make up of the catchment populations and service delivery across the three sites (total population 4.2 million). Ethical approval was independently gained from the West Midlands Research Ethics Committee (WMREC Ref 08/H1208/44) and Birmingham City Council Ethics Committee.

### Sample size and power calculation

A pilot study indicated that two to three MHA assessments occured every day in each participating Trust. It was anticipated that about 675 assessments would occur over a three month period (average 2.5/day/trust) across the three sites. The aim was to collect data on all MHA assessments during the study periods, however, assuming that complete data would be available for about 60% of assessments, this gave a tentative sample size of 405 assessments for each three month period. This sample size had the power to detect a 10% difference in detention rates with 85% power and 5% significance. The study periods were later extended to four months.

### Case identification

A MHA assessment was defined as any clinical encounter where an Approved Social Worker (ASW, as defined in the MHA 1983) or an Approved Mental Health Professional (AMHP, as defined in the MHA 2007) were involved or where at least one medical recommendation for detention had been completed, regardless of the outcome of the assessment (i.e. detention, voluntary admission or no admission). In order to identify all MHA assessments, researchers made weekly contact with clinical teams, including on-call clinicians and crisis resolution/home treatment teams at each site, to identify all MHA assessments conducted in the previous week. Cross-checks were made with the dataset of MHA assessments held by Social Services in order to maximise identification of all assessments.

### Data collection

Data were collected from local forms used for MHA assessment documentation and patient electronic databases. The data collection occurred over four-month periods across four years: July to October in the years 2008 (under the MHA 1983), 2009 (within a year of implementation of the MHA 2007), 2010 and 2011 at all study sites. Before starting data collection, researchers arranged meetings with ASWs/AMHPs to describe the study, answer any queries and to request that the local forms used for MHA data collection (CR6B and SS101) were completed with as much detail as possible.

The CR6B form provided information on details of previous admissions to hospital and the circumstances leading to the latest assessment/reassessment. It provided a record of interviews and discussions with the patient, Nearest Relative and others, and with assessing doctors and other professional staff. The discussion included consideration of appropriate medical treatment, including options of alternative provision of care, any Advance Directives by the service user and the assessment of risk to the service user and others. The service user’s social situation, including details of accommodation, employment and family and social relationships, were also recorded on this section. The SS101 section of the form recorded demographic information about and the contemporaneous legal status of the service user under the MHA, the location of the assessment and the outcome of assessment, i.e. whether or not the service user was detained under the Act. Information from the CR6B/SS101s was collated and encoded at each site using PASW Statistics version 18 [7].

Patient electronic databases (i.e. EPEX, Rio and Jade) at participating Mental Health Trusts were also utilised to maximise the quality of data collection. Data entry was completed at all sites by the end of March 2012. Subsequently a combined database of all assessments was made using SPSS v19 [8].

### Diagnoses

The above data sources were scrutinised to identify diagnoses and, where available, ICD 10 codes [9]. If ICD 10 codes were not specified, F codes (e.g. F20 Schizophrenia) or blocks (e.g. F20-F29 Schizophrenia, schizotypal and delusional disorders) were allocated from available information. A series of diagnosis variables were therefore re-coded for some cases for the purposes of this paper, e.g. the MHA assessment of a patient diagnosed with a right hemispheric organic affective disorder, where no ICD F code was noted in the social care or mental health documentation, would have a variable noting the stated diagnosis (*right hemispheric organic affective disorder*); another for the F code (*F07.8* denoting Other organic personality and behavioural disorders due to brain disease, damage and dysfunction); another denoting the ICD 10 Chapter V block (*F00-F09* denoting Organic, including symptomatic, mental disorders); and yet another variable of relevance to the research question (i.e. the F code being allocated to a category of *personality disorders*). For a minority of assessments where this process could not be followed, diagnosis was classified by information available (e.g. *comorbid diagnosis* recorded, but the exact diagnoses were not). The assessment outcome variable was also re-cleaned, with unclear outcomes (e.g. outcome marked as “no bed available”) recoded as missing data.

### Statistical analysis

Descriptive statistics were created for the sociodemographic and clinical characteristics of the of the patients assessed. These were proportions and percentages for categorical data; means and standard deviations for continuous data. Fisher’s Χ2 tests were carried out to test for associations between variables of interest to explore any differences between the detained population before (2008) and after (2009–11) the amendments to the Act. Where small samples were available, two sided Fisher’s exact tests were used wherever possible. Both the Χ2 statistic and significant associations at the 5% level are reported.

Missing Data: Whilst all efforts were made to retrieve data where possible, some information remained unknown. Throughout this paper, cases with missing entries in any variable under consideration are excluded from that analysis (i.e. complete case analysis).

## Results

There were 4423 MHA assessments across all three sites through the four study periods: 1273 in Birmingham, 1104 in Oxford and 2046 in London. Table 1 gives the breakdown of assessments at each site by the year and outcome of assessment, patient age and gender. In these assessments, 48.7% of patients were already under a Section 2 (detention for assessment and possible treatment up to 28 days), Section 3 (detention for treatment up to six months), Section 4 (detention for assessment and possible treatment up to 72 h), Section 5 (s5(2)- a doctor’s emergency holding power for assessment under the Act for up to 72 h; s5(4)- a nurse’s emergency holding power for up to 6 h), Section 7 (Guardianship obliging the patient to reside in a particular place), Section 135 (warrants under the Act for assessments on private premises or to recover patients who are absent without leave) or Section 136 (police power to detain someone in immediate need of care or control and remove them to a place of safety, lasting up to 72 h) (see Table 2).

**Table 1:** MHA assessments at each site by the year of assessment; patient age; gender and outcome of the assessment

		Birmingham *N* (%)	Oxford *N* (%)	London *N* (%)	All sites *N* (%)
Year of assessment	2008	256 (20.1)	292 (26.6)	500 (24.4)	1048 (23.7)
	2009	353 (27.7)	213 (19.4)	503 (24.6)	1069 (24.2)
	2010	402 (31.6)	308 (28.1)	569 (27.8)	1279 (23.1)
	2011	262 (20.6)	283 (25.8)	474 (23.2)	1019 (23.1)
	Missing	0	8	0	8
Age group	Under 30	328 (26.6)	310 (28.2)	487 (24.2)	1125 (25.9)
	30 and over	904 (73.4)	790 (71.8)	1525 (75.8)	3219 (74.1)
	Missing	41	4	34	79
Gender	Male	744 (58.5)	569 (51.5)	1184 (58.0)	2497 (56.5)
	Female	527 (41.5)	535 (48.5)	857 (42.0)	1919 (43.5)
	Missing	2	0	5	7
Outcome of assessment	Detained	746 (60.1)	640 (59.1)	1485 (73.8)	2871 (66.2)
	Not detained	495 (39.9)	442 (40.9)	527 (26.2)	1464 (33.8)
	Missing	32	22	34	88

**Table 2:** Legal status of patient at assessment

Legal status category	Number of assessments	Percent	Valid percent
Community/None	1504	34.0	40.9
Hospital informal	192	4.3	5.2
Section 2	347	7.8	9.4
Section 3	173	3.9	4.7
Section 4 or 5	309	7.0	8.4
Section 7	148	3.3	4.0
Section 135 or 136	813	18.4	22.1
Other (includes CTO^a^)	193	4.4	5.2
Missing	744	16.8	

^a^Community Treatment Order

Table 3 gives details of the diagnosis at assessment by ICD Chapter 5 groups. The most common group was schizophrenia, schizotypal and delusional disorders (F20-F29), followed by mood/affective disorders (F30-F39), with much smaller numbers for other categories. In 62 assessments a diagnosis was given, but not classified as mental or behavioural. E.g. epilepsy. In 343 assessments, no diagnosis was recorded or no mental illness was found. Multiple mental and behavioural diagnoses were given in 174 assessments and the diagnosis was missing in 290 assessment records.

**Table 3:** Diagnosis at assessment by ICD Chapter 5 block

Diagnosis category	Frequency within mental and behavioural diagnoses	Percent
Organic, including symptomatic, mental disorders (F0-F09)	109	3.1
Mental and behavioural disorders due to psychoactive substance use (F10-F19)	144	4.1
Schizophrenia, schizotypal and delusional disorders (F20-F29)	1812	51.0
Mood/ affective disorders (F30-F39)	889	25.0
Neurotic, stress-related and somatoform disorders (F40-F48)	50	1.4
Behavioural syndromes associated with physiological disturbances and physical factors (F50-F59)	32	0.9
Disorders of adult personality and behaviour (F60-F69)	248	4.2
Mental retardation (F70–79)	11	0.3
Disorders of psychological development (F80-F89)	56	1.6
Behavioural and emotional disorders with onset usually occurring in childhood and adolescence (F90-F98)	10	0.3
Unspecified mental disorder (F99)	193	5.4
Total	3554	

Of the 4274 assessments where an outcome was noted, 1499 (33.9%) resulted in detention under Section 2; 1349 (30.5%) in detention under Section 3 and 55 (1.2%) in a Guardianship order. Of the 4274, the result was informal admission in 319 (7.2%) assessments, informal community treatment in 442 (10.0%) assessments and no psychiatric intervention in 405 (9.2%) of assessments. Of the remaining 205 (4.6%) other outcomes from the 4274 assessments, 12 (0.3%) were revokations of Community Treatment Orders. There were nine forensic detentions in the dataset (detention under sections 35, 36, 37, 37/41, 38, 47 or 48), all of which occurred after the changes to the MHA (2009 and 10).

### Did the number of individuals being assessed and detained change after implementation of the MHA 2007?

The total number of assessments in each four month period of data collection varied: 1034 in 2008, 1042 in 2009, 1242 in 2010 and 1010 in 2011 (*n* = 4415). The reciprocal numbers of detentions were 678, 743, 804 and 641. 65.6% of assessments resulted in detention in 2008, 71.3% in 2009, 64.7% in 2010 and 63.5% in 2011. There was no significant change in the odds ratio of detention when comparing the 2008 assessments against the combined 2009, 2010 and 2011 data (OR = 1.025, Fisher‘s exact *Χ* ^*2*^ *p* = 0.735).

### Did the MHA 2007 lead to more detentions for patients with personality disorders, sexual deviance, learning and developmental disabilities?

There were no assessments of patients labelled as having disorders such as paedophilia, sadomasochism, voyeurism, sexual exhibitionism, fetishism, fetishistic transvestism, multiple disorders of sexual preference, other disorders of sexual preference, disorder of sexual preference not specified or labels akin to them that would fall under F65 (disorders of sexual preference). Table 4 presents the numbers and proportions of assessed cases with PD, disorders of psychological development (including pervasive developmental disorders), disorders usually occurring in childhood and adolescence, mild to profound mental retardation and cases that would fall under a remnant category of ‘any other disorder or disability of the mind’ (usually coded as F99 ‘Unspecified mental disorder’ or ‘other’). Only patients with mild to profound mental retardation and any other disorder or disability of the mind were significantly more likely to be assessed under the MHA post implementation (Χ^2^ = 5.485, *P* = 0.018; Χ^2^ = 24.962, *P* > 0.001 respectively).

**Table 4:** Numbers of assessed patients with diagnoses of interest

Diagnosis at assessment	Year of assessment n (% of assessments with valid diagnoses in that timeframe)			Significance
	2008	2009–11	All	
Personality disorder (F60-F69 and F07). This includes Disorders of sexual preference (F65) covering paedophilia, sadomasochism, voyeurism, sexual exhibitionism, fetishism, fetishistic transvestism, multiple disorders of sexual preference, other disorders of sexual preference and disorder of sexual preference not specified.	57 (6.2)	258 (8.1)	315 (7.6)	0.058
Mental retardation (F70–79)	1 (0.1)	26 (0.8)	27 (0.6)	0.018
Disorders of psychological development (F80-F89, includes Specific developmental disorders of speech and language, motor skills, scholastic skills (e.g. Specific spelling disorder) and Pervasive developmental disorders, i.e. autism spectrum disorders)	16 (1.7)	57 (1.8)	73 (1.8)	1
Disorders usually occurring in childhood and adolescence (F90-F98)	3 (0.3)	13 (0.4)	16 (0.4)	1
Any other disorder or disability of the mind (F99 Unspecified mental disorder or “other”)	25 (2.7)	230 (7.2)	255 (6.2)	>0.001

Of the 308 PD cases where the outcome of assessment was known, 23/57 were detained in 2008 and 125/251 were detained in 2009–11 but there was no significant change in the proportions of patients detained (Χ^2^ = 1.662, *p* = 0.240) before or after the changes to the MHA. A similar pattern is seen for patients with diagnoses of disorders of psychological development (8/15 detained in 2008; 26/54 detained 2009–11; Χ^2^ = 0.126, *p* = 0.78) and patients with diagnoses of disorders usually occurring in childhood and adolescence (2/3 detained in 2008; 6/13 detained 2009–11; Χ^2^ = 0.410, *p* = 1).

One patient with mental retardation was assessed and detained in 2008; 19/24 were detained 2009–11; but there was no significant change in the proportions detained Χ^2^ = 0.260, *p* = 1. Similarly 17/25 with any disorder or disability of the mind were detained in 2008; 142/223 detained 2009–11; Χ^2^ = 0.126, *p* = 0.78.

## Discussion

Our main findings were that both the numbers of assessments and of detentions increased in the first two years after implementation of the MHA 2007 but fell in the third year. The rates of detention, as a proportion of assessments, remained about the same. There were no assessments or detentions of patients with sexual deviance. There was a significant increase in the proportions of patients assessed under the MHA who had mental retardation or who could be labelled as having ‘any other disorder or disability of the mind’ after implementation of the 2007 Act. There was no significant change in the proportions of patients detained with PD, LD or disorders usually occurring in childhood and adolescence. These findings indicate that the underlying aim of facilitating the compulsory care of patients with PDs, sexual deviance and LDs was not achieved in the first three years post-implementation of the MHA 2007.

Many socio-political and service provision-related factors can act to change the way the MHA is used or revised, complicating the interpretation of data of this nature [10]. For example, although rates of detention have been rising for a number of years, the overall rate of admission for mental disorders has fallen as community-based alternatives have been provided; reduction of in-patient beds within the NHS has, however, been closely associated with an increasing rate of detention to NHS hospitals since the 1980s, especially when analysis includes a time lag of one year between bed provision and subsequent involuntary admissions [11]. Public and government concern about limiting risks posed by dangerous individuals have even produced new categories of mental disorder, such as Dangerous Severe Personality Disorder, without any other theoretical or scientific pedigree [12, 13].

Legal and ethical issues can influence research methodology. For example, the data on multiple assessments for the same patient can not be collated when a study such as AMEND is on MHA assessments and not individuals. This is because ethics and data protection law-related requirements prevent social care and mental health provider organisations passing patient identifiable details to research teams. A secondary analysis of assessments of the same patient is therefore precluded by research teams’ inability to cross check data from the two types of service (social care collecting data on assessments and mental health trusts on patients and their care).

### Strengths and limitations of the study

The sample size calculation for this study was robust. Expected numbers of assessments per day were consistent with published data [14].

There can be variation between geographical areas and service provision. Analysis, however, of anonymised records of over 1.2 million people for 2010/11 held in the Mental Health Minimum Data Set (the mandatory return from providers of National Health Service-funded mental health care in England), Census data and data from commissioning and provider healthcare organisations indicated that 85% of the variance in detention rates occurred between individuals; only around 7% of statistically significant variance occurred between places (Census areas) and around 7% again between provider healthcare organisations [15]. There was also heterogeneity in the socio-economic and ethnic make up of the catchment populations and service delivery across the three sites, making the findings of AMEND nationally generalisable.

The AMEND study only covered a limited period between 2008 and 2011. We do not know how representative the 2008 data was of assessments undertaken in previous years.

Although multiple sources for data collection were used and cross-checked, it is possible that some MHA assessments were missed. Given that not all assessments had all of their specific subcategories of diagnosis recorded, some groups (e.g. personality disorders) are only approximations of categories of interest. Approximately 70 cases were excluded from the analysis as the immediate outcome of the assessment was not clear from the notations from the database. Given the small numbers of forensic detentions, all of which occurred after the 2007 Act was implemented, conclusions could not be drawn about whether there was shift between forensic and civil sections.

### Comparison with other studies

Other post-legislative reports on the MHA 2007 do not present detailed data on assessments or diagnoses of assessed or detained patients [16–18]. Government data from the years leading up to implementation of the 2007 Act is available on formal admissions and detentions subsequent to admission under the MHA. Data includes detentions after being subject to a place of safety order, detentions in NHS facilities and independent hospitals. In 2006–07, 2007–08 and 2008–09 numbers detained were 46,539, 47,610 and 47,725 respectively [19].

The pattern in the AMEND data of an increase in assessments and detentions from 2008 to 2010 and the subsequent decrease in 2011 is difficult to explain. When we compare AMEND data with Government data, we need to remember that the AMEND data was collected July to October in the years of 2008–2011 and Governmental data is collected for years running from 1st April to 31st March for each data year. The latter also does not give data on the numbers of assessments, collect data from Child and Adolescent Mental Health Services (CAMHS) or Learning Disability Services or provide information on diagnoses but did indicate that the total number of detentions (excluding Guardianship) increased by 13% over the five years from 2008 to 9 to 2012–13 [20]. An interesting observation is that in the third year post implementation of the MHA 1983 there was also a slight dip in rates of detention in the context of year by year increases in numbers of detention from from 1984 to 1991 [10].

Data from the Mental Health Minimum Data Set is difficult to compare with AMEND data as the former provides information on the number of adults of working age and people over the age of 65 years who spent time formally detained in an NHS hospital under the MHA [21]. Each person is counted only once and in the category with the most restrictive legal status that applied to them in the year. The focus on detained patients makes invisible those who are assessed but not detained. The only other large-scale study of MHA assessments in England was published a quarter of a century ago and reported data collected in 1984 by the Social Services Research Group to monitor the implementation of the MHA 1983 [22].

### Implications for research and clinical practice

Despite the importance, good quality research and interpretation of data in this area is difficult because of the large number of explanatory, confounding and biasing variables. This in turn means that adequately powered studies, collecting data on the appropriate range of individual, socio-political, professional, service- and practice-related variables will be required. Governmental monitoring of the use of the MHA, e.g. through the Care Quality Commission, should also make visible the needs and experiences of those who are assessed but not detained, as their health and rights are also affected by implementation of the Act.

AMEND, unlike parallel Government data, did not collect information from private sector (independent) hospitals. Government data [20] shows that although NHS hospital detentions in England increased from 2008 to 9 (with the exception of 2011–12) to 2012–13 (25,185, 27,475, 26,937, 27,855 and 28,779 respectively), there was a sharper rise in detentions to independent hospitals from 2011 to 12 (2761, 2712, 2620, 3045 and 3445 respectively). By the data year 2012–13, independent providers were responsible for the care of just over a quarter of people detained under the MHA. Some of the main independent providers are not however submitting all records pertaining to detentions and hence the significance of private provision may be rising even faster [20]. Future research should be just as assiduous in collecting data from independent as NHS providers.

It will be important to enquire why the MHA 2007 may not have had the hoped for impact. Perhaps it reflects a lack of knowledge of the MHA amongst relevant professionals [23] or the individual policies and practices of relevant agencies [22]. Perhaps the role that capacity plays in decisions about whether to treat people against their will needs further enquiry [24] as although people with personality disorder may experience high levels of mental distress, loss of capacity is rare [25] and the majority of mental health practitioners have been shown to be concerned about detaining people with personality disorder against their will [26]. Another possible explanation is a consequence of the very common association between serious personality disorder and severe mental illness, a combination very likely to be associated with compulsory admission [27]; In practice clinicians may ignore the personality disorder when making a diagnosis of mental illness. The diagnosis of personality disorder then remains covert even though it is one of the main drivers of coercion when detaining a patient under the Act. It is likely that qualitative studies, such as AMEND’s parallel enquiry into mental health professionals’ understanding and interpretation of the Appropriate Treatment Test, will be needed to add to our understanding of the reasons why changes to the 1983 Act appear to have had such little impact on clinical practice.

Future research in this area could also benefit from the additional use of validated, structured clinical interview schedules to establish the presence or absence of specific diagnoses rather than using approximations of diagnostic categories. This would help in any comparison of practice in differing jurisdictions, however, we acknowledge that there would be significant ethical, practical procedural challenges that would make such research very difficult to execute and complete.

## Conclusions

AMEND is the only large-scale study of Mental Health Act assessments in England in a quarter of a century. It shows that, in the three years following implementation of the MHA 2007, the underlying aim of facilitating the compulsory care of patients with PDs, sexual deviance and LDs was not achieved.

## Abbreviations

AMHP: Approved Mental Health Professional
ASW: Approved Social Worker
LDs: learning disabilities
MHA: Mental Health Act
NHS: National Health Service
PDs: personality disorders
